# Immune and Metabolic Dysregulated Coding and Non-coding RNAs Reveal Survival Association in Uterine Corpus Endometrial Carcinoma

**DOI:** 10.3389/fgene.2021.673192

**Published:** 2021-06-24

**Authors:** Da Liu, Min Qiu

**Affiliations:** ^1^Department of Obstetrics and Gynecology, Shengjing Hospital of China Medical University, Shenyang, China; ^2^Department of Orthopedics, Shengjing Hospital of China Medical University, Shenyang, China

**Keywords:** dysregulated network, endometrial carcinoma, miRNA, lncRNA, integrative analysis, TCGA, immunity, metabolism

## Abstract

Uterine corpus endometrial carcinoma (UCEC) is one of the most common gynecologic malignancies, but only a few biomarkers have been proven to be effective in clinical practice. Previous studies have demonstrated the important roles of non-coding RNAs (ncRNAs) in diagnosis, prognosis, and therapy selection in UCEC and suggested the significance of integrating molecules at different levels for interpreting the underlying molecular mechanism. In this study, we collected transcriptome data, including long non-coding RNAs (lncRNAs), microRNAs (miRNAs), and messenger RNAs (mRNAs), of 570 samples, which were comprised of 537 UCEC samples and 33 normal samples. First, differentially expressed lncRNAs, miRNAs, and mRNAs, which distinguished invasive carcinoma samples from normal samples, were identified, and further analysis showed that cancer- and metabolism-related functions were enriched by these RNAs. Next, an integrated, dysregulated, and scale-free biological network consisting of differentially expressed lncRNAs, miRNAs, and mRNAs was constructed. Protein-coding and ncRNA genes in this network showed potential immune and metabolic functions. A further analysis revealed two clinic-related modules that showed a close correlation with metabolic and immune functions. RNAs in the two modules were functionally validated to be associated with UCEC. The findings of this study demonstrate an important clinical application for improving outcome prediction for UCEC.

## Introduction

Cancer is one of the major public health problems worldwide and is the second leading cause of death in the United States (Siegel et al., 2021). After the rapid development in healthcare, the total decline in the cancer death rate has reached approximately 31% (Siegel et al., 2021). Nonetheless, uterine corpus endometrial carcinoma (UCEC) is still one of the most common gynecologic malignancies in many countries (Matteson et al., 2018). In the United States alone, there will be approximately 14,000 new UCEC patients and 4,000 deaths in the 2021, as predicted by Siegel et al. (Siegel et al., 2021). Generally, UCEC is prevalent among postmenopausal women due to the unstable level of estrogen (Chen et al., 2015). Different risk factors, such as smoking, high blood pressure, and being overweight, also contribute to the generation and development of UCEC (Zhang et al., 2014). In particular, changes in molecular levels are one factor contributing the development of UCEC (Li et al., 2020). However, effective therapeutic targets are still scarce in clinical practice.

Non-coding RNAs (ncRNAs), including microRNAs (miRNAs) and long non-coding RNAs (lncRNAs), have been regarded as transcriptional noise and useless due to their low effective transcription and expression (Hyashizaki, 2004). Taking advantage of the large-scale, next-generation transcriptomic sequencing, more ncRNAs have been identified. In GENCODE v29, there are 16,066 annotated lncRNA genes, 7,577 annotated small ncRNA genes (e.g., miRNA) and thousands of other ncRNA genes. In total, there are more than 30,000 annotated ncRNA genes, which are more than protein-coding genes whose annotated number is less than 20,000. Many ncRNAs have been functionally associated with human diseases, such as cancers (Gutschner and Diederichs, 2012). HOX antisense intergenic RNA (HOXAIR), one of the most famous lncRNAs, has been reported to be associated with metastases in colorectal, liver, pancreatic, breast, and gastric cancers (Gupta et al., 2010; Kogo et al., 2011; Yang et al., 2011). Furthermore, some ncRNAs have been functionally related with UCEC. Wang found a six-miRNA signature that can predict the survival of UCEC patients (Wang et al., 2019). Many studies have investigated the pathogenesis at genomic levels using the combination of different kinds of molecules and have discovered clinical diagnostic and prognostic biomarkers. It reported that miR-21 and lncRNA AWPPH are associated with the poor prognosis of hepatocellular carcinoma but regulate cancer cell chemosensitivity and proliferation in triple-negative breast cancer (Liu et al., 2019). Dong et al. revealed two patient survival-associated RNA sets, including lncRNAs, miRNAs, and messenger RNAs (mRNAs), in invasive breast carcinoma (Dong et al., 2020). Moreover, Liu et al. identified six triplets of mRNA–lncRNA–miRNA that play a function in UCEC (Liu et al., 2017) based on the expression profiles. However, their underlying molecular mechanisms still need to be uncovered.

In this study, to investigate the underlying molecular mechanisms of the generation and development of UCEC, the expression profiles of 537 UCEC and their 33 counterpart normal samples were downloaded from the Cancer Genome Atlas (TCGA). Three different kinds of RNAs, namely, lncRNAs, miRNAs, and mRNAs, were extracted from the profiles. First, a differential expression analysis was performed, followed by a functional enrichment analysis, including a gene ontology (GO) analysis, KEGG analysis, and gene set enrichment analysis (GSEA). Then, a lncRNA–miRNA–mRNA dysregulated network was constructed, and two modules related with the survival time, metabolic function, and immune function were identified. RNAs from each module have showed a functional role in UCEC.

## Materials and Methods

### Acquisition of RNA Sequencing Datasets

RNA sequencing datasets of 570 samples were downloaded from TCGA^1^, including 537 UCEC samples and 33 normal samples (Supplementary Table 1). Each sample contained miRNAs, lncRNAs, and mRNAs simultaneously were used for downstream analyses. The annotation from GENCODE database (GENCODE v36) was used to extract lncRNAs from the expression profile. Based on the annotation file, the following biotypes were regarded as known lncRNAs: antisense, lincRNA, lncRNA, processed_transcript, sense_intronic, sense_overlapping, and TEC. The biotype “protein_coding” was used to extract mRNAs from the expression profile. Finally, 19,597 mRNAs, 15,088 lncRNAs, and 188 miRNAs were used for the downstream analysis.

### Differential Expression Analysis

To remove biases, RNAs with an expression level in less than 10% of the samples were ignored, followed by a differential expression analysis with *p*-value < 0.05 and fold change > 2 using a *t*-test (Ye et al., 2018). In total, 648 differentially expressed lncRNAs, 5,831 differentially expressed mRNAs, and 342 differentially expressed miRNAs were identified (Supplementary Table 2). Unsupervised clustering was performed, and heat maps were drawn for differentially expressed lncRNAs, mRNAs, and miRNAs using the R package pheatmap, separately. Moreover, the R package Prcomp was used to conduct the principal component analysis (PCA).

### MiRNAs and Their Targets

MiRNA target sites were downloaded from one of the most popular databases in the field, starBase v3.0 (Li et al., 2014), which predicts the miRNA target using five algorithms, i.e., TargetScan (Lewis et al., 2005), miRanda (Enright et al., 2003), Pictar2 (Krek et al., 2005), PITA (Kertesz et al., 2007), and RNA22 (Loher and Rigoutsos, 2012). MiRNAs play a function in RNA-induced silencing complexes (RISCs), or the ribonucleoprotein complexes (Fabian et al., 2010). The components of RISCs, i.e., Argonaute (AGO) family proteins, are the best characterized protein elements and are central to RISC functions (Chekulaeva and Filipowicz, 2009). Ultraviolet (UV) crosslinking and immunoprecipitation (CLIP) is one of the useful techniques in identifying specific protein–RNA interactions, including identifying the AGO–RNA–miRNA complex to illustrate miRNA functions (König et al., 2012). Thus, in this study, AGO CLIP-Seq datasets downloaded from starBase v3.0 were used to identify AGO binding sites. MiRNA-target pairs with at least one AGO binding site were considered. Finally, 40,042 miRNA–lncRNA and 1224,551 miRNA–mRNA regulatory relationships were used, which include 3,228 lncRNAs, 413 miRNAs, and 14,645 mRNAs.

### Functional Enrichment Analysis

To explore the functional roles of differentially expressed molecules, GO and KEGG analyses were performed using clusterProfiler (Yu et al., 2012). For ncRNAs, we first calculated the Pearson correlation coefficient between each ncRNA-mRNA pair based on the expression value across the samples, followed by the calculation of the average Pearson correlation coefficient for each mRNA across ncRNAs. Then, the top 500 co-expressed mRNAs were used. Barplots were drawn using ggplot2. To further investigate the functional roles of the key RNAs, GSEA was also performed using clusterProfiler (Yu et al., 2012).

To determine if genes in each immune (or metabolism)-related pathway are enriched in each sample, the Gene Set Variation Analysis (GSVA) (Hänzelmann et al., 2013) was performed. Gene sets annotated in immune (or metabolism)-related pathways were obtained from MSigDB^2^. GSVA scores were calculated using the R package GSVA with the single-sample GSEA method.

### Construction of the Dysregulated lncRNA–miRNA–mRNA Network

First, the miRNA–lncRNA and miRNA–mRNA interactions from starBase v3.0 (Li et al., 2014) were obtained. Only differentially expressed miRNAs, lncRNAs, and mRNAs were considered for the downstream analysis. Then, the dysregulated lncRNA–miRNA–mRNA network was constructed based on the interactions. Afterward, a two-step filtering was used: (1) The correlations between each miRNA-target pair should be significant (*p*-value < 0.01 and | correlation coefficient| > 0.3) across all samples using the Pearson correlation coefficient. (2) Only miRNAs shared by mRNAs and lncRNAs were used. Finally, a dysregulated network was constructed containing 1243 interactions, including 323 mRNAs, 52 miRNAs, and 53 lncRNAs (Supplementary Table 3). To identify functional modules, CytoCluster (Li et al., 2017), a graphical algorithm, was used with the hierarchical clustering algorithm in protein interaction networks (HC-PIN) and default parameters. CytoCluster is a Cytoscape plugin integrating six clustering algorithms, i.e., identifying overlapping and hierarchical modules in protein interaction networks (OH-PIN), identifying protein complex algorithm (IPCA), clustering with overlapping neighborhood expansion (ClusterONE), detecting complexes based on an uncertain graph model (DCU), identifying protein complexes based on maximal complex extension (IPC-MCE), and the Biological Networks Gene Ontology (BinGO) function. CytoCluster is a very popular algorithm used to identify functional modules, predict protein complexes and network biomarkers, and visualize clustering results.

### Survival Analysis

The clinical data of all the UCEC and normal samples were obtained from TCGA, and the survival time was extracted using a customized Perl script. For each module, the samples were clustered into two different groups via k-means clustering based on the expression across the RNAs, followed by the comparison of the survival durations between the two groups using a log-rank test. Finally, an R package survival was used to conduct the statistical test.

## Results

### Dysregulated RNAs Can be Used to Distinguish UCEC Samples From Normal Ones

The expression profiles of 570 samples for miRNAs, lncRNAs, and mRNAs were downloaded from TCGA, which include 537 UCEC samples and 33 counterpart normal samples (Supplementary Table 1). To investigate the underlying molecular mechanism on how UCEC occurs and develops, a differential expression analysis was performed for each expression profile using a *t*-test with a *p*-value < 0.05 and fold change > 2 as the cutoff (see section “Materials and Methods”). A total of 5831 differentially expressed mRNAs between the UCEC and normal samples were identified, which include 2810 upregulated and 3021 downregulated genes (Supplementary Table 2). Moreover, 648 differentially expressed lncRNAs were identified, including 219 upregulated and 428 downregulated lncRNAs (Supplementary Table 2). We also identified 342 differentially expressed miRNAs, in which 280 were upregulated and 62 were downregulated (Supplementary Table 2).

**FIGURE 1:**
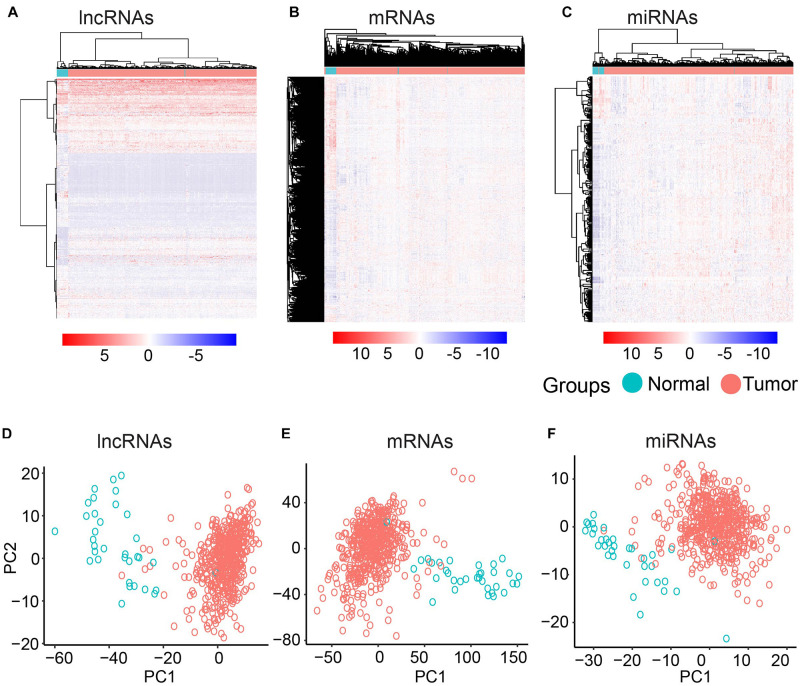
Clustering based on differentially expressed molecules. Heatmap of clustering for UCEC and the normal samples based on differentially expressed lncRNAs **(A)** mRNAs **(B)** and miRNAs **(C)**. PCA analysis for differentially expressed lncRNAs **(D)** mRNAs **(E)** and miRNAs **(F)**.

To further investigate the differentially expressed mRNAs, lncRNAs, and miRNAs between the UCEC and their counterpart normal samples, an unsupervised hierarchical clustering analysis was performed using the R package pheatmap. Each molecule can clearly distinguish UCEC samples from their counterpart normal samples (Figures 1A–C). Furthermore, PCA was conducted for the differentially expressed lncRNAs, mRNAs, and miRNAs using the R function prcomp. Again, the majority of the UCEC samples and their counterpart normal samples were separated into two groups (Figures 1D–F).

**FIGURE 2:**
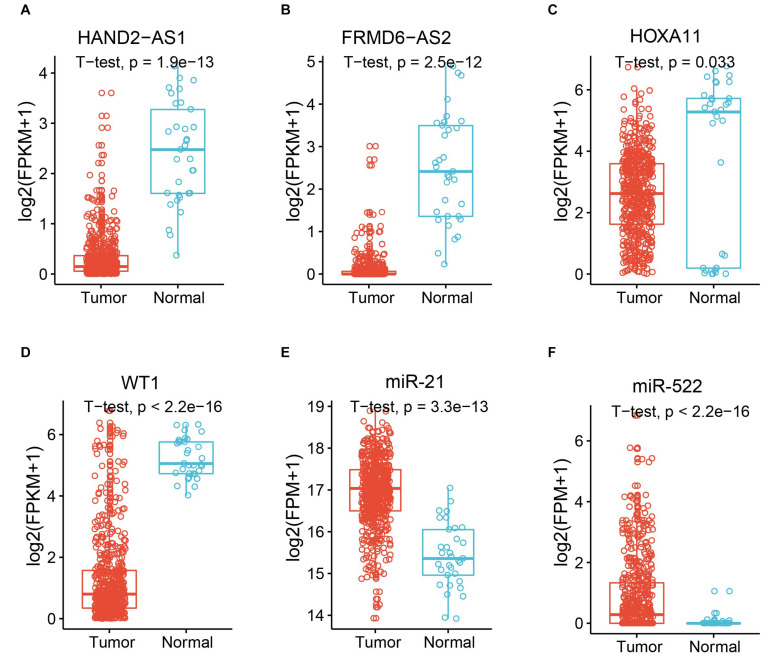
Expression of example molecules in UCEC and the normal samples. The comparison of gene expression between tumor sample and the normal sample for differentially expressed lncRNAs HAND2-AS1 **(A)** and FRMD6-AS2 **(B)**, differentially expressed genes HOXA11 **(C)** and WT1 **(D)**, and differentially expressed miRNAs miR-21 **(E)** and miR-522 **(F)**.

The known tumor suppressor lncRNA HAND2 Antisense RNA 1 (HAND2-AS1) was identified as one of the differentially expressed lncRNAs in high-grade serous ovarian carcinoma (Yang et al., 2018). The significant downregulation in UCEC indicated the potential role as a tumor suppressor in UCEC (Figure 2A). Another lncRNA example is FRMD6 Antisense RNA 2 (FRMD6-AS2), which is also downregulated in UCEC (Figure 2B). Wang et al. reported the tumor suppressive effect of this lncRNA in UCEC, whose expression is consistent here (Wang et al., 2020). For the protein-coding gene, Homeobox protein Hox-A11 (HOXA11) was significantly downregulated in UCEC (Figure 2C) and was reported to play roles in malignant cancer (Zhang et al., 2018). WT1 was also downregulated in UCEC (Figure 2D), which was reported to be a prognostic marker in advanced serous epithelial ovarian carcinoma (Netinatsunthorn et al., 2006). MicroRNA-21 (miR-21), which was upregulated in UCEC (Figure 2E), is also a cancer biomarker (Bautista-Sánchez et al., 2020). The suppression role for the proliferation and metastasis of miR-522 in non-small cell lung cancer was reported by Zhang et al. (2016), in which miR-522 was upregulated in UCEC (Figure 2F). All these data indicate the potential functional roles of these key RNA molecules.

### Dysregulated Genes Are Highly Enriched in Cancer- and Metabolism-Related Pathways

**FIGURE 3:**
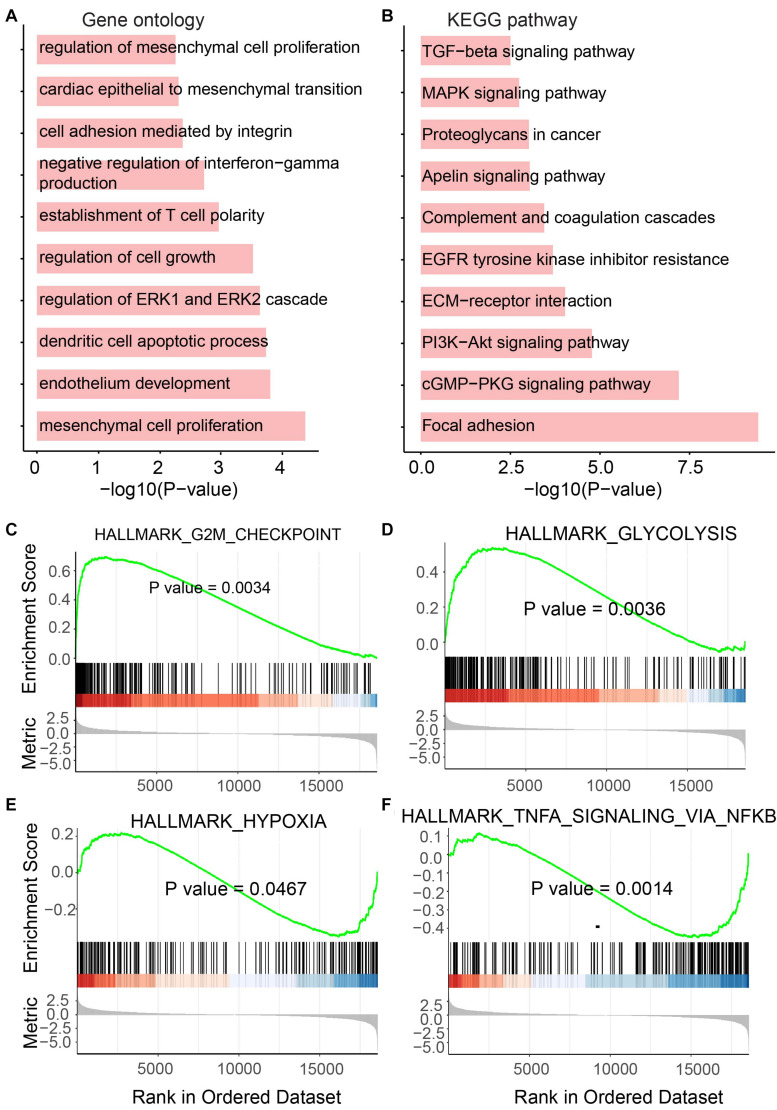
Functional enrichment analysis for differentially expressed mRNAs. **(A)** Enriched GO terms. **(B)** Enriched KEGG pathways. **(C–F)** Results of GSEA analysis.

As we mentioned above, genes playing an important function in tumor generation and development were identified to be up- or downregulated in UCEC. To determine the functional roles for all differentially expressed mRNAs, an unbiased functional enrichment analysis for GO using clusterProfiler (Yu et al., 2012) was performed. Cancer hallmark-related terms were enriched (Figure 3A). Apoptotic processes, such as “dendritic cell apoptotic process,” and cell proliferation-related pathways, such as “mesenchymal cell proliferation” and “regulation of mesenchymal cell proliferation,” were enriched. Moreover, immunity-related terms were enriched, such as “establishment of T-cell polarity.”

A functional enrichment analysis for KEGG was also performed by the UCEC-related genes (Figure 3B). Phosphatidylinositol-4,5-bisphosphate 3-kinase (PI3K)/protein kinase B (Akt) pathway, which is associated with cellular quiescence, proliferation, cancer, and longevity, is an intracellular signaling pathway of great importance in the cell cycle process. It was enriched by UCEC-related genes. The pathway “proteoglycans in cancer” was also enriched, which suggested the functional roles of differentially expressed mRNAs in cancer.

To further investigate the roles of these UCEC-related genes, GSEA was performed using clusterProfiler (Yu et al., 2012; Figures 3C–F). The glycolytic pathway, whose high level in tumors, including UCEC, exhibits specific driver genes in most cancer types (Wei et al., 2020), was enriched by upregulated genes in UCEC (Figure 3D). Upregulated genes in UCEC were also enriched in a hypoxia-related pathway (Ruan et al., 2009; Figure 3E). Moreover, known tumor-related pathways, i.e., G2M checkpoint (Figure 3C) and TNFA (Figure 3F) related terms, were enriched by up- and downregulated genes, respectively.

### Dysregulated ncRNAs Reveal Immune and Metabolic Functions

**FIGURE 4:**
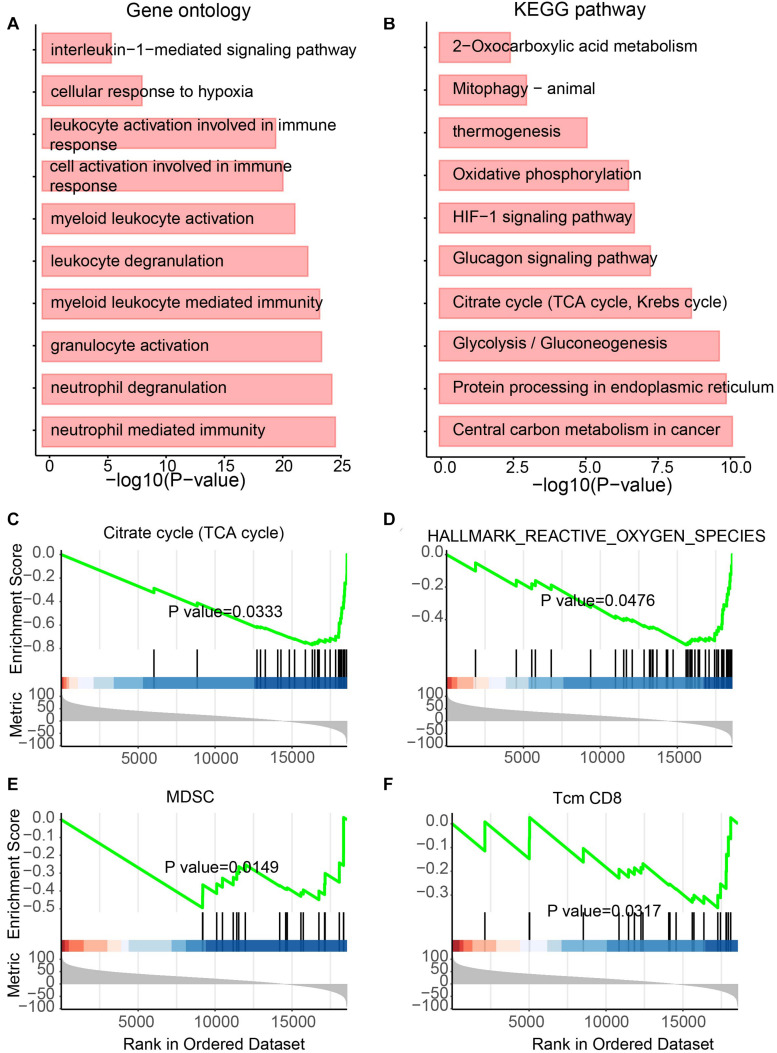
Functional enrichment analysis for differentially expressed lncRNAs. **(A)** Enriched GO terms. **(B)** Enriched KEGG pathways. **(C–F)** Results of GSEA analysis.

NcRNAs have previously been regarded as useless for a long time. However, recently, more studies have attempted to explore the function of ncRNAs (Jiang et al., 2019) and showed functional ncRNAs in tumors (Dong et al., 2020). To determine the functional roles of differentially expressed lncRNAs in UCEC, GO and KEGG analyses were performed (Figures 4A,B). For the GO analysis, immunity-related terms, such as “neutrophil-mediated immunity,” “neutrophil degranulation,” “myeloid leukocyte-mediated immunity,” “leukocyte degranulation,” “myeloid leukocyte activation” and “interleukin-1-mediated signaling pathway” were enriched (Figure 4A). For the KEGG analysis, metabolic pathways, such as “central carbon metabolism in cancer,” “glycolysis/gluconeogenesis,” “glucagon signaling pathway,” “oxidative phosphorylation,” and “thermogenesis” were enriched by these lncRNAs (Figure 4B).

In addition, to further identify the roles of these lncRNAs, GSEA was also performed (Figures 4C–F). Metabolic features, such as “TCA cycle,” “Hallmark reactive oxygen species pathway,” and “myeloid-derived suppressor cell” were enriched (Figures 4C–E). The immunity-related feature “T-cell memory (Tcm) CD8” was also enriched (Figure 4F). Interestingly, all these features were enriched by downregulated lncRNAs in UCEC, suggesting the immune and metabolic functional roles of these downregulated lncRNAs.

**FIGURE 5:**
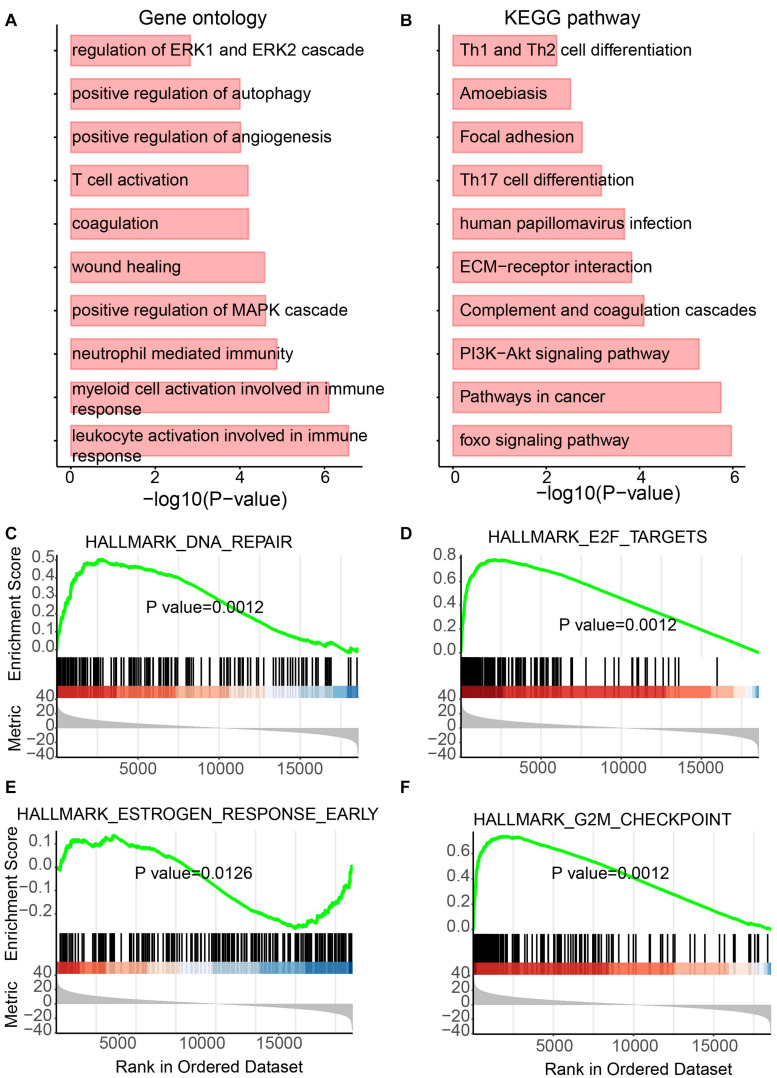
Functional enrichment analysis for differentially expressed miRNAs. **(A)** Enriched GO terms. **(B)** Enriched KEGG pathways. **(C–F)** Results of GSEA analysis.

Besides lncRNAs, miRNAs were also reported to play essential roles in tumor development (Qiu et al., 2020). Thus, to determine the functional role of differentially expressed miRNAs, the same analyses performed on lncRNAs were performed for miRNAs. Again, metabolism and immunity-related GO terms and KEGG pathways were enriched (Figures 5A,B). Metabolic GO terms, such as “positive regulation of MAPK cascade” and “regulation of ERK1 and ERK2 cascade,” and immunity-related terms, such as “leukocyte activation involved in immune response,” “myeloid cell activation involved in immune response” and “neutrophil-mediated immunity” were enriched. Similarly, GSEA also showed the enrichment of pathways involving in cancer and metabolic diseases (Figures 5C–F). The DNA repair pathway, which has been reported to be the target for cancer therapies (Helleday et al., 2008) and plays roles in metabolic diseases (Hoeijmakers, 2009), was enriched by upregulated miRNAs in UCEC (Figure 5C). The E2F pathway was also enriched by upregulated miRNAs in UCEC (Figure 5D). E2F plays a key role in tumor suppression through a specific regulation of tumor suppressor genes (Kurayoshi et al., 2018). Furthermore, estrogen-related and G2M pathways were enriched by downregulated and upregulated miRNAs in UCEC, respectively (Figures 5E,F). Estrogens show function in controlling the energy balance and glucose homeostasis (Mauvais-Jarvis et al., 2013) and play roles in different cancer types (Whiteside, 2008).

### Construction of the Dysregulated lncRNA–miRNA–mRNA Network

**FIGURE 6:**
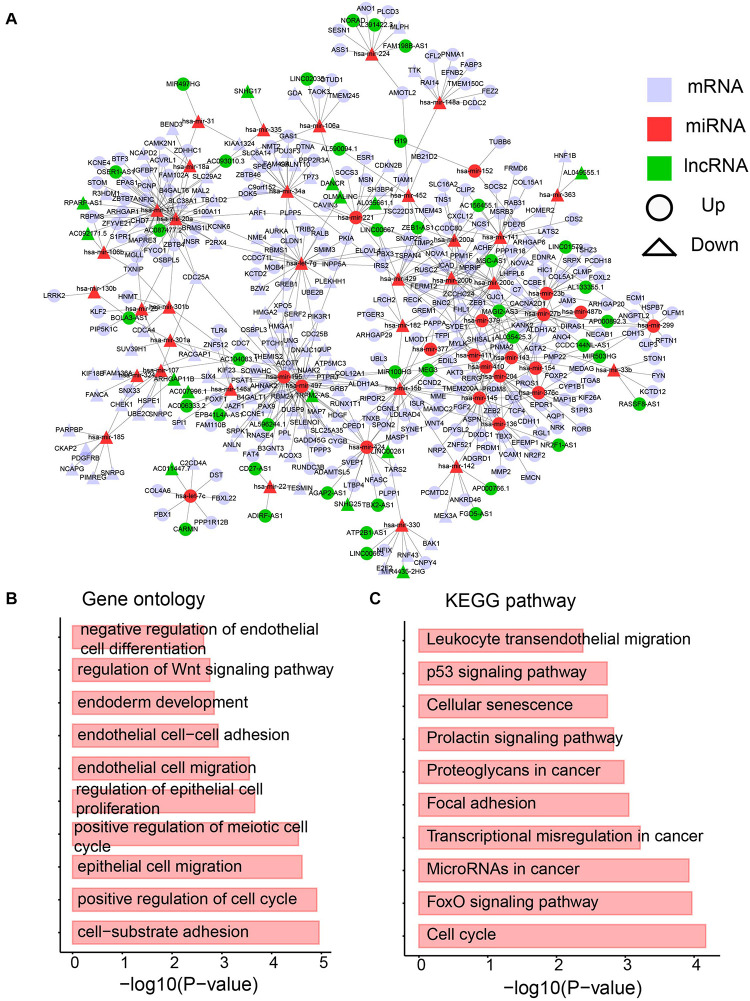
The dysregulated lncRNA-miRNA-mRNA network. **(A)** The network containing differentially expression mRNA, lncRNA and miRNA. **(B)** Enriched GO terms. **(C)** Enriched KEGG pathways.

Based on the interactions between miRNA and its targets downloaded from starBase v3.0 (Li et al., 2014), a dysregulated network containing differentially expressed lncRNAs, miRNAs, and mRNAs was constructed. To provide more confident interactions between miRNA and its targets, AGO CLIP-Seq was used, followed by several filtering steps (see section “Materials and Methods”). A final dysregulated lncRNA–miRNA–mRNA network was constructed with 1243 interactions and 428 differentially expressed molecules, including 323 mRNAs, 53 miRNAs, and 53 lncRNAs (Figure 6A and Supplementary Table 3).

A biological network is a small-world network (Latora and Marchiori, 2001) or scale-free network (Latora and Marchiori, 2001). To test whether our dysregulated network is a scale-free network, the degree distribution was analyzed (Supplementary Figure 1). Approximately 90% of RNAs have less than five edges, whereas only approximately 5% of RNAs have more than 10 interactions. The data support that our dysregulated network is a scale-free network and a meaningful biological network. To further investigate the network, a GO analysis was performed. Cancer hallmark-related functions were enriched, such as the migration-related term “epithelial cell migration” and proliferation-related term “regulation of epithelial cell proliferation” (Figure 6B). Moreover, pathways involved in the metabolism were enriched (Figure 6B). The Wnt signaling pathway has been shown to direct glycolysis and angiogenesis in colon cancer (Pate et al., 2014). In addition, the KEGG pathway analysis was performed. Pathways playing function in cancers, such as “proteoglycans in cancer,” “microRNAs in cancer” and “transcriptional misregulation in cancer” were enriched by the differentially expressed RNAs in the dysregulated network (Figure 6C). The FoxO pathway was also enriched (Figure 6C), which was reported to be a therapeutic target in cancers (Farhan et al., 2017) and regulate glucose and lipid metabolism (Lee and Dong, 2017). All these data imply the immune and metabolic functions of our dysregulated network.

### The Dysregulated Networks Showed Clinical-Related Modules

**FIGURE 7:**
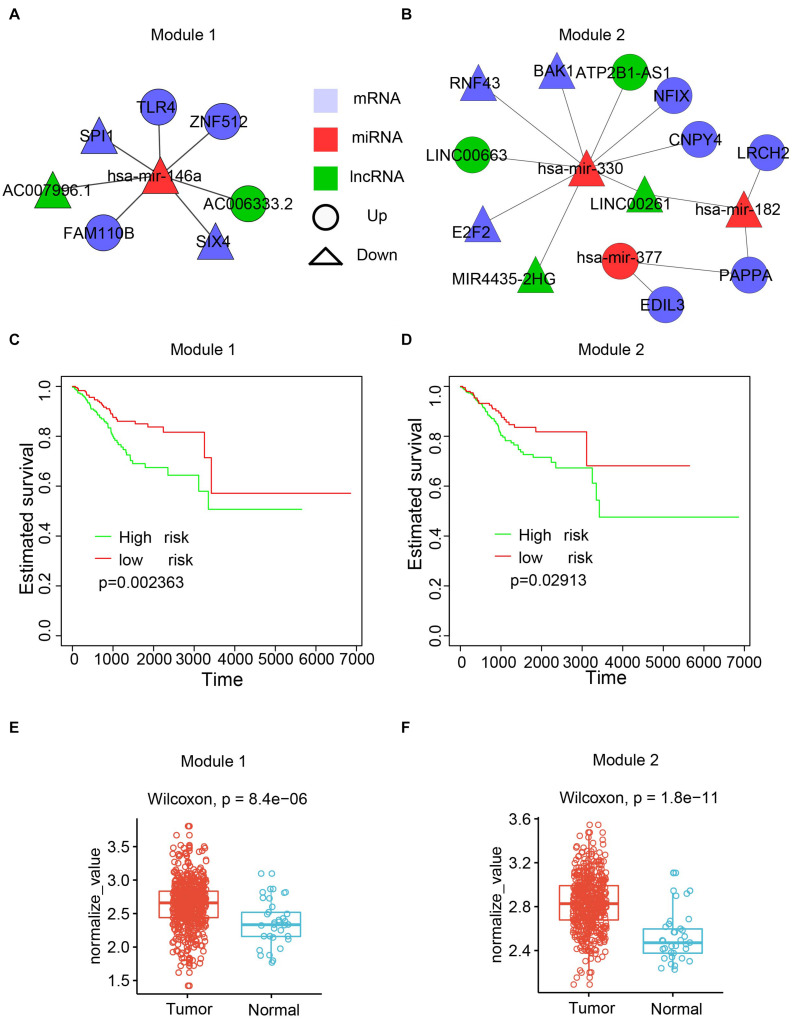
Functional modules identified from the dysregulated network. **(A)** The first module. **(B)** The second module. **(C)** Kaplan-Meier plot of survival for the first module. **(D)** Kaplan-Meier plot of survival for the second module. **(E)** Expression patterns of the first modules in normal and cancer samples. The average expression value of each molecule crossing all normal/cancer samples was used. **(F)** Expression patterns of the second modules in normal and cancer samples.

To maximize the utility of the dysregulated lncRNA–miRNA–mRNA network, the Cytoscape plugin CytoCluster (Li et al., 2017) was used to identify functional modules from the dysregulated network. CytoCluster is a popular tool used to identify functional modules by integrating seven clustering algorithms, namely, HC-PIN (Wang et al., 2011), OH-PIN (Wang et al., 2012), IPCA (Li et al., 2008), ClusterONE (Nepusz et al., 2012), DCU (Zhao et al., 2014), IPC-MCE (Li et al., 2010), and BinGO function. Accordingly, two modules were identified (Figures 7A,B). The first module contained 7 interactions with 5 mRNAs, 2 lncRNAs, and 1 miRNA. The second one consisted of 14 interactions with 8 mRNAs, 4 lncRNAs, and 3 miRNAs.

To explore the biological function of the two modules, the associations of the modules with the patient survival time were evaluated by checking the difference of the survival time between two subpopulations from all UCEC patients divided by the k-means clustering. Both modules showed a significant correlation with the survival time (Figures 7C,D). Next, the Wilcoxon rank-sum test was performed based on the expression values of RNAs between the tumor and normal samples. The results showed that both modules had higher expression in the UCEC samples compared with their counterpart normal samples (Figures 7E,F).

### The Clinical-Related Modules Are Correlated With Metabolism and Immunology

As immunity- and metabolism-related functions were connected to the dysregulated RNAs in the network, we focused on these related pathways. To determine if the dysregulated RNAs in the two modules are correlated with the immune and metabolic functions, GSVA (Hänzelmann et al., 2013) was performed for each sample. GSVA provides increased power to detect subtle pathway activity changes over a sample population in comparison to corresponding methods.

**FIGURE 8:**
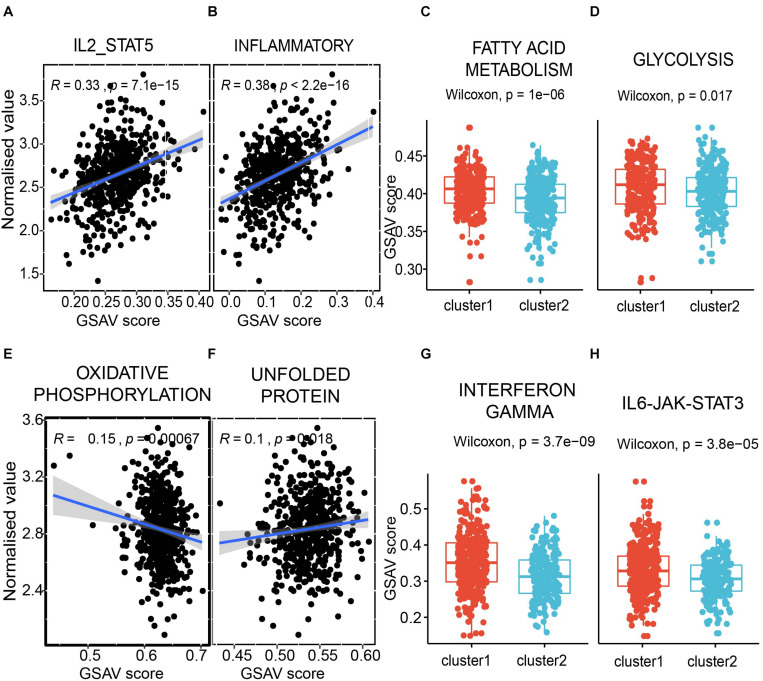
The modules correlated with immunity and metabolism. **(A,B)** the correlation between the first module and pathway IL2-STAT5 **(A)**, and inflammatory **(B)**. Each dot presents a sample. X axis presents the GSVA score, and Y axis presents the normalized expression value. The average expression value of the first module for each sample was used. **(C,D)** the comparison between two subpopulations from Figure 7C in fatty acid metabolism pathway **(C)** and glycolysis pathway **(D)**. Y axis presents the GSVA score. **(E,F)** the correlation between the second module and pathway oxidative phosphorylation **(E)**, and unfolded protein **(F)**. **(G,H)** the comparison between two sub-populations from Figure 7D in interferon gamma pathway **(G)** and IL6-JAK-STAT3 pathway **(D)**.

The first module is positively correlated with interleukin-2 and STAT5 pathway (Figure 8A), which was reported to regulate T-cell development and function (Mahmud et al., 2013). A known immune inflammatory pathway was also positively correlated in the first module (Figure 8B). Furthermore, two classical metabolic pathways, i.e., fatty acid metabolism pathway and glycolysis pathway, showed significantly different GSVA scores between the two subpopulations with different survival times in the module shown in Figures 7C, 8C,D. The same analyses were also performed to the second module. Oxidative phosphorylation, a classic metabolic pathway, showed a negative correlation with the second module (Figure 8E). The unfolded protein pathway, which showed functional roles in different cancer types (McGrath et al., 2018) and metabolic pathways (Lee and Ozcan, 2014), was positively correlated with the second module (Figure 8F). The interferon gamma pathway, which affects tumor progression and regression in different cancers (Jorgovanovic et al., 2020) and also metabolic signalings (Siska and Rathmell, 2016), showed significantly different GSVA scores between the two subpopulations with different survival times in the second module shown in Figures 7D, 8G. A similar scenario occurred in the IL6/JAK/STAT3 pathway, a well-known pathway playing a significant role in cancers (Johnson et al., 2018; Figure 8H).

**FIGURE 9:**
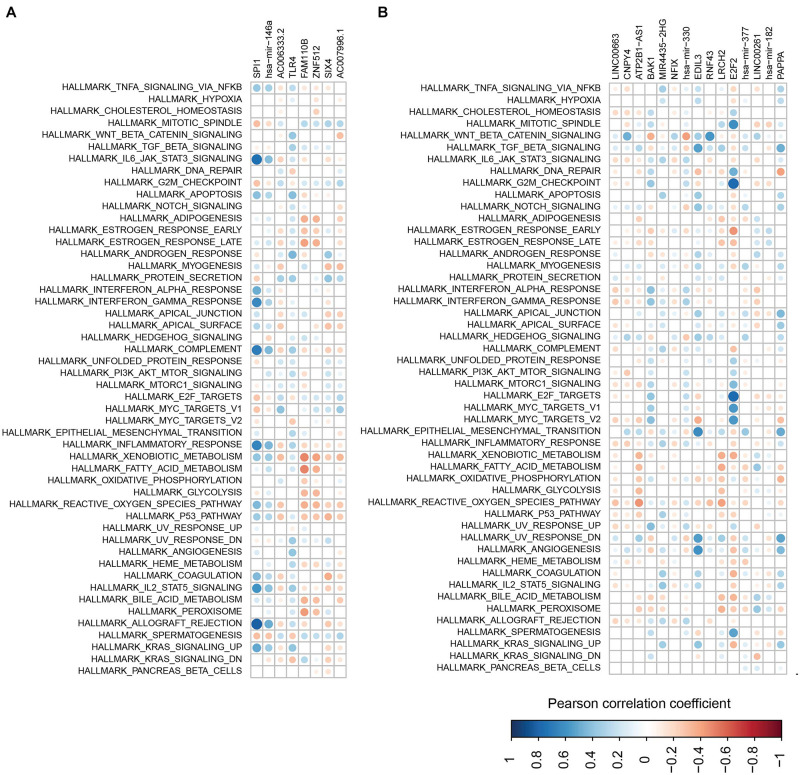
The correlations between RNAs and pathways. **(A,B)** Correlations between pathway and RNAs from the first module **(A)** and the second module **(B)**.

To further check the function of the two modules, the correlation between each RNA in the modules and the pathways involved in the immune and metabolic functions was examined (Figures 9A,B). Overall, SP11, miR-146a, AC006333.2, and TLR4 from the first module showed a negative correlation with the metabolic and immune functions (Figure 9A). Conversely, the other four RNAs in the first module more likely have a positive correlation with the metabolic and immune functions. In the second module, several RNAs, especially for E2F2, showed a negative correlation with the metabolic and immune functions (Figure 9B). E2F2 was highly negatively correlated with pathways involved in G2M checkpoints, E2F targets, and mitotic spindles.

## Discussion

In this study, a dysregulated lncRNA–miRNA–mRNA network was constructed, in which all RNAs were differentially expressed in UCEC and enriched in cancer and metabolic functions. An integrative analysis on transcriptome data from 570 samples was performed at three different RNA levels, i.e., lncRNAs, miRNAs, and mRNAs. Further analysis identified two clinical-related modules, which showed correlation with metabolic and immune functions. Importantly, some elements from the two modules have been functionally related with UCEC. This framework will help reveal the underlying mechanism for the generation and development of UCEC.

NcRNAs, which constitute more than 90% of RNAs made from the human genome, have attracted increasing attention as more ncRNAs have been functionally validated in different conditions, particularly in human diseases, such as cancers (Anastasiadou et al., 2017; Slack and Chinnaiyan, 2019). In this study, to better determine the potential roles of ncRNAs in UCEC, we focused on dysregulated lncRNAs and miRNAs. By taking advantage of state-of-the-art technologies, we integrated dysregulated lncRNAs, miRNAs, and mRNAs into a single dysregulated network, which is a scale-free and biologically meaningful network. Based on the dysregulated lncRNA–miRNA–mRNA network, a functional enrichment analysis for GO and KEGG was performed, and the results showed that metabolic and immune functions that the network may be involved in were enriched.

Further analysis identified two modules including dysregulated lncRNAs, miRNAs, and mRNAs using a Cytoscape plugin CytoCluster. By integrating the corresponding clinical data, we found that the two modules were survival time related, and both modules were overexpressed in the UCEC samples, indicating the potential carcinogenic roles of some overexpressed elements in the two modules. Through GSVA, we further showed that both modules were immunity and metabolism related. Nevertheless, the biggest limitation is that all the conclusions were drawn without any experiments to support them. Although some elements in the two modules have been functionally validated in UCEC, there are genes (i.e., TLR4, FAM110B, LINC00663, and LINC00261) in the two modules that have not been reported in UCEC, and further experimental and clinical validations are necessary for these RNAs with potential functional roles in UCEC. In the future, we would select one of the genes for further investigation. The counterpart functional experiments such as knockdown and overexpression assays to investigate the mechanism on how the gene paly function in UCEC would be performed. Our study provides new insights into the outcome prediction and will help in the precision medicine for UCEC.

## Data Availability Statement

The original contributions presented in the study are included in the article/Supplementary Material, further inquiries can be directed to the corresponding author/s.

## Ethics Statement

The patient data used in this study was acquired as publicly available datasets that were collected with patients’ informed consent.

## Author Contributions

MQ conceived the project. MQ and DL collected the data and reviewed the manuscript. DL performed analysis and wrote the manuscript. Both authors contributed to the article and approved the submitted version.

## Conflict of Interest

The authors declare that the research was conducted in the absence of any commercial or financial relationships that could be construed as a potential conflict of interest. The handling editor declared a past co-authorship with the authors MQ and DL.

## Footnotes

1 https://portal.gdc.cancer.gov/

2 http://www.gsea-msigdb.org/gsea/msigdb
