# Supplementary figures and images for: Immune and Metabolic Dysregulated Coding and Non-coding RNAs Reveal Survival Association in Uterine Corpus Endometrial Carcinoma
Source: Front Genet. 2021 Jun 24;12:673192. doi: 10.3389/fgene.2021.673192 (PMC8264798; doi:10.3389/fgene.2021.673192)

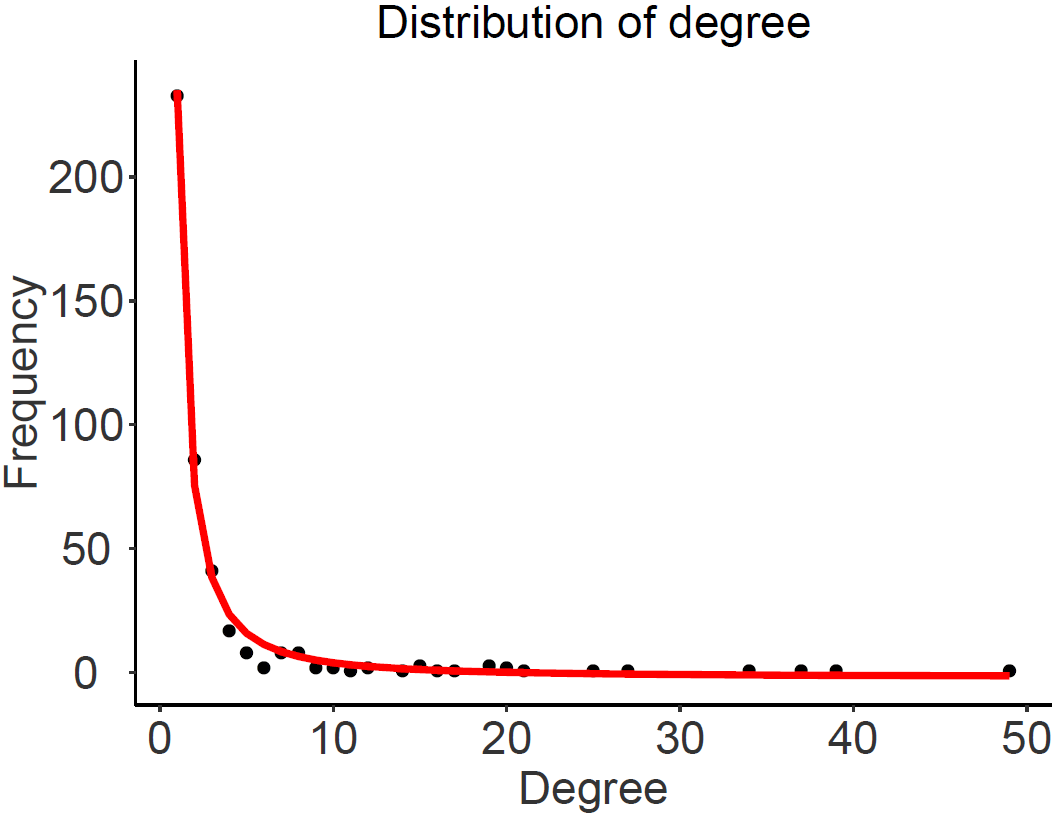


**Figure S1. The distribution of degree**.

Supplement: Supplementary file 1 [file Table_1.DOCX]
